# Transcriptomic analysis identifies the shared diagnostic biomarkers and immune relationship between Atherosclerosis and abdominal aortic aneurysm based on fatty acid metabolism gene set

**DOI:** 10.3389/fmolb.2024.1365447

**Published:** 2024-04-10

**Authors:** Xuefeng Gu, Zhongxian Yu, Tianwei Qian, Yiqi Jin, Guoxiong Xu, Jiang Li, Jianfeng Gu, Ming Li, Ke Tao

**Affiliations:** ^1^ Department of General Surgery, Changshu Hospital Affiliated to Soochow University, Changshu, Jiangsu Province, China; ^2^ Department of General Surgery, The Affiliated Suzhou Hospital of Nanjing Medical University, Suzhou, Jiangsu Province, China

**Keywords:** abdominal aortic aneurysm, bioinformatics, Atherosclerosis, lipid metabolism disorder, diagnostic biomarker

## Abstract

**Background:**

Epidemiological research has demonstrated that there is a connection between lipid metabolism disorder and an increased risk of developing arteriosclerosis (AS) and abdominal aortic aneurysm (AAA). However, the precise relationship between lipid metabolism, AS, and AAA is still not fully understood. The objective of this study was to examine the pathways and potential fatty acid metabolism-related genes (FRGs) that are shared between AS and AAA.

**Methods:**

AS- and AAA-associated datasets were retrieved from the Gene Expression Omnibus (GEO) database, and the limma package was utilized to identify differentially expressed FRGs (DFRGs) common to both AS and AAA patients. Functional enrichment analysis was conducted on the (DFRGs), and a protein-protein interaction (PPI) network was established. The selection of signature genes was performed through the utilization of least absolute shrinkage and selection operator (LASSO) regression and random forest (RF). Subsequently, a nomogram was developed using the results of the screening process, and the crucial genes were validated in two separate external datasets (GSE28829 and GSE17901) as well as clinical samples. In the end, single-sample gene set enrichment analysis (ssGSEA) was utilized to assess the immune cell patterns in both AS and AAA. Additionally, the correlation between key crosstalk genes and immune cell was evaluated.

**Results:**

In comparison to control group, both AS and AAA patients exhibited a decrease in fatty acid metabolism score. We found 40 DFRGs overlapping in AS and AAA, with lipid and amino acid metabolism critical in their pathogenesis. PCBD1, ACADL, MGLL, BCKDHB, and IDH3G were identified as signature genes connecting AS and AAA. Their expression levels were confirmed in validation datasets and clinical samples. The analysis of immune infiltration showed that neutrophils, NK CD56dim cells, and Tem cells are important in AS and AAA development. Correlation analysis suggested that these signature genes may be involved in immune cell infiltration.

**Conclusion:**

The fatty acid metabolism pathway appears to be linked to the development of both AS and AAA. Furthermore, PCBD1, ACADL, MGLL, BCKDHB, and IDH3G have the potential to serve as diagnostic markers for patients with AS complicated by AAA.

## Introduction

Abdominal aortic aneurysm (AAA) is a cardiovascular condition characterized by the abnormal enlargement of the aorta, exceeding 50% of its normal diameter. This condition can potentially result in the rupture of the aorta, leading to severe bleeding (Sakalihasan et al., 2018). Typically, patients with AAA do not experience any symptoms until a rupture occurs. This is a prevalent and potentially fatal illness that results in over 150,000 deaths worldwide every year (Golledge et al., 2006; Ullery et al., 2018). Aneurysms exceeding 5.5 cm in diameter, which expand quickly within a brief timeframe and disrupt blood flow to organs far from the heart, may be suitable for traditional open surgery or endovascular repair of the aorta. Nonetheless, relying solely on these size parameters as indicators does not yield highly accurate assessments (Chaikof et al., 2018). Moreover, during the monitoring period, small AAA may burst, and currently, there is an insufficient range of effective treatments to alter the course of AAA development (Golledge, 2019; Oliver-Williams et al., 2019). Hence, it is essential to unravel the fundamental processes driving the initial advancement of AAA in order to pinpoint precise targets for effective treatment.

Atherosclerosis (AS), a condition characterized by the thickening of arterial walls as a result of the accumulation of lipids and inflammation, is affected by the equilibrium between inflammatory and reparative mechanisms (Bäck et al., 2019). The onset of this disease occurs when lipoproteins penetrate the arterial lining and are taken up by macrophages, resulting in the formation of fatty foam cells. Insufficient removal of these cells exacerbates the advancement of the condition (Sukhorukov et al., 2020; Cui et al., 2021). The growing body of evidence indicates a potential link between AS and AAA (Trollope and Golledge, 2011; Peshkova et al., 2016; Hołda et al., 2020; Takahashi, 2021). The pathways associated with the metabolism of lipids could potentially elucidate the shared pathogenic mechanism between AS and AAA. AS can begin with the accumulation of Apo B-containing lipoproteins within the walls of arteries, which leads to the activation of the endothelium and the influx of monocytes. This process contributes to the buildup of cells, extracellular matrix, and lipids within the arterial vessels (Ruparelia and Choudhury, 2020). In individuals with AS, disturbances in the metabolism of liver lipids could heighten the likelihood of developing atherosclerotic conditions (Zhang et al., 2022). In addition, imbalanced lipid metabolism, which causes irregular cholesterol levels, is a key risk factor for AS. Consequently, therapies focusing on restoring lipid homeostasis are essential in reducing AS incidents (Aguilar-Ballester et al., 2020). The experimental induction of AAA leads to significant changes in the metabolic composition of both aortas and blood. These changes are primarily focused on the disruption of nitric oxide production, lipid metabolism, and energy-related metabolic pathways (Guo et al., 2020; Chao de la Barca et al., 2022). Elevated lipoprotein levels have been found to be an independent indicator of increased risk of disease in patients with AAA (Kubota et al., 2018). In patients with AAA, there is a notable elevation in low-density lipoprotein, which primarily transports cholesterol produced within the body. This increase is considered a potential risk factor that could adversely affect the outlook for individuals with AAA (Hobbs et al., 2003). Conversely, high-density lipoprotein, which primarily facilitates the removal of cholesterol from the bloodstream, exhibits an inverse relationship with the risk of AAA (Takagi et al., 2010). These findings implied a significant correlation between lipid metabolism, AS, and AAA. Yet, the exact molecular mechanism and the pathological correlations are still not well-defined. Therefore, investigating biomarkers associated with lipid metabolism could be immensely valuable for comprehending the underlying mechanisms and developing future treatments for individuals suffering from AS who are further complicated by AAA.

Based on this foundation, our proposed approach involves utilizing bioinformatics to analyze datasets related to AS and AAA in the GEO database. The aim is to identify fatty acid metabolism-related genes (FRGs), and validate their differential expression using external datasets and clinical samples. Additionally, we conducted single-sample gene set enrichment analysis (ssGSEA) to evaluate the immune cell patterns in AS and AAA. Furthermore, we calculated the correlation between key shared genes and each immune cell type. The ultimate goal is to gain new insights into the mechanisms underlying the onset and progression of AS and AAA, and to identify potential biomarkers for the diagnosis and treatment of individuals with AS complicated by AAA.

## Methods

### Raw data acquisition and pre-processing

Gene expression profiles for AS and AAA were sourced from the Gene Expression Omnibus (GEO), accessible at the following URL: https://www.ncbi.nlm.nih.gov/geo/. The selection of datasets included the following criteria: (i) the generation of gene expression profiles via array techniques; (ii) arterial tissue served as the source for sample collection; (iii) the inclusion of datasets comprising a minimum of 10 samples; (iv) the availability of raw data for investigative purposes. After applying these parameters, the datasets GSE57691 (AAA), GSE47472 (AAA), GSE98278 (AAA), GSE17901 (AAA), GSE100927 (AS), and GSE28829 (AS) were identified and chosen for subsequent analysis. Supplementary Table S1 displays the characteristics of the participants. The affy package was utilized to carry out preprocessing and normalization of these datasets. Due to the limited number of samples in the datasets related to AAA, especially with the control group having fewer than 10 samples, the combat function from the sva package was used to integrate three datasets (GSE57691, GSE47472, and GSE98278) associated with AAA. As a result, there were 18 control samples and 94 AAA samples in total. This combined dataset is used as the analysis set, defined as AAA-related merged data sets. To validate the signature genes implicated in AAA, we employed the GSE17901 dataset, which is derived from a mouse model using ApoE−/− mice subjected to angiotensin II-induced AAA (Spin et al., 2011). This well-characterized model was chosen due to its documented mimicry of human AAA pathology, particularly in terms of vascular inflammation and immune response. GSE100927 includes 35 control samples and 69 AS samples, while GSE28829 consists of 13 early AS and 16 advanced AS (Table 1). In addition, 158 FRGs was gathered from the Molecular Signatures Database (MSigDB).

**TABLE 1:** Summary of GEO datasets involving AS and AAA patients.

AAA-related datasets	GEO ID	Platform	Control group	Disease group	Source	Application	Species
	GSE57691	GPL10558	10	49	Aortic wall	Analysis	*Homo sapiens*
	GSE47472	GPL10558	8	14	Aortic wall	Analysis	*Homo sapiens*
	GSE98278	GPL10558	0	31	Aortic wall	Analysis	*Homo sapiens*
	GSE17901	GPL4134	6	7	Suprarenal aorta	Validation	*Mus musculus*
AS-related datasets	GSE100927	GPL17077	35	69	Arterial tissue	Analysis	*Homo sapiens*
	GSE28829	GPL570	13 (early AS)	16 (advanced AS)	Arterial tissue	Validation	*Homo sapiens*

### Identifying differentially expressed genes (DEGs)

We utilized the limma package to conduct a comparative analysis of gene expression between the control (Con) and disease groups. The DEGs were identified by applying the criterion of a p. adj <0.05 (Yuan et al., 2022). Volcano maps were utilized to visualize DEGs from datasets, with the assistance of the ggplot package.

### Assessment of fatty acid metabolism score between con and disease groups

The fatty acid metabolism score was evaluated using the gene set variation (GSVA) approach. To achieve this, we utilized the GSVA package to compute the fatty acid metabolism score for each sample, based on the fatty acid metabolism gene set. The obtained scores were then graphically displayed using a violin diagram.

### Identifying differentially expressed FRGs (DFRGs)

To acquire the expression profiles of DFRGs, we cross-referenced the identified FRGs with DEGs from both the integrated AAA dataset and the GSE100927 dataset. Utilizing the pheatmap package, we constructed a heatmap to depict the expression patterns of these DFRGs.

### Establishment of a network illustrating interactions between proteins

We employed the Search Tool for the Retrieval of Interacting Genes (STRING) database (available at http://string-db.org/) to analyze protein-protein interactions (PPI) among the identified overlapping DFRGs. Only PPI with a confidence score exceeding 0.7 were considered. The resulting interaction network was then visualized using Cytoscape software (version 3.9.0). To further explore the biological functions of DFRGs, we utilized the clusterProfiler package for Kyoto Encyclopedia of Genes and Genomes (KEGG) pathway analysis and Gene Ontology (GO) enrichment analysis.

### Machine learning identifies signature genes

The Least Absolute Shrinkage and Selection Operator (LASSO) is a regression method that is employed for regularization in order to enhance the accuracy of predictions and improve the comprehensibility of the model (Li et al., 2022). We employed the glmnet package to conduct LASSO regression analysis in order to identify the most effective predictors for AS and AAA within the aforementioned DFRGs. DFRGs were utilized as input to construct a Random Forest (RF) model by employing the randomForest package in R, utilizing a RF classifier. We determined the significance levels of gene variables using the MeanDecreaseGini method. Genes that exhibited an importance value of one or higher were deemed critical and subsequently selected for the next phases of model development and validation. For the diagnosis of AS and AAA, the signature genes were pinpointed as the common genes shared by both LASSO and RF methods.

### Development and evaluation of a nomogram model

To create the nomogram, we employed the rms package in R, considering the selected candidate genes. A specific score, known as “points,” was allocated to each of these genes. The “Total Points” signifies the aggregate score derived from the entire set of genes. The effectiveness of the nomogram in diagnosing AS and AAA was determined by constructing a Receiver Operating Characteristic (ROC) curve. Additionally, the precision of the model was evaluated by examining calibration curves and employing decision curve analysis (DCA).

### GeneMANIA database

GeneMANIA (genemania.org) is a user-friendly web platform that aids in understanding gene functions, analyzing gene groups, and choosing genes for tests. It predicts functionally similar genes using genomic and proteomic data upon user input (Franz et al., 2018). GENEMANIA was utilized to construct a network of interactions among genes to evaluate the functions of key signature genes.

### Immune microenvironment analysis

The ssGSEA algorithm, which incorporates gene sets linked to a diverse range of immune cell types, functions, checkpoints, and pathways, was employed to thoroughly assess the immunological characteristics inherent to each sample (Mooney et al., 2013). Using ssGSEA, we quantified the infiltration of 23 different immune cell types in both control and diseased tissue samples. We then employed the pheatmap package to create a heatmap that illustrates the varying patterns of immune cell infiltration across these samples. The ggplot2 package was used to perform a detailed analysis of the relationship between the expression levels of diagnostic genes, the fatty acid metabolism score, and the degree of immune cell infiltration.

### Verification of the signature genes expression by clinical samples

Samples of arterial tissue were obtained from the popliteal arteries of 8 AS patients at Changshu Hospital Affiliated to Soochow University. During open surgical procedures for repairing full-thickness abdominal aortic aneurysms, tissue samples from 8 AAA were collected, while tissue from six healthy visceral aortas was sourced from organ donors at the time of kidney transplants taking place at Changshu Hospital Affiliated to Soochow University. Before collecting data, we obtained written consent from all participants and received approval for the study protocol from the Ethics Committee of Changshu Hospital Affiliated to Soochow University. Supplementary Table S2 displays the baseline characteristics of the participants.

The TRIzol reagent (Invitrogen, Thermo Fisher) was used to process tissue samples for RNA extraction, following the instructions provided by the manufacturer. Quantitative real-time PCR (qRT-PCR) analysis was performed using the SYBR qPCR Master Mix (Bio-Rad) after reverse transcription of total RNA to cDNA with cDNA synthesis kits (Invitrogen, Thermo Fisher). We utilized the Roche LC480 Real-Time PCR System to quantify the expression levels of the signature genes. The internal control for mRNA, GAPDH, was employed in this study.

## Results

### Genetic screening for differential analysis

**FIGURE 1:**
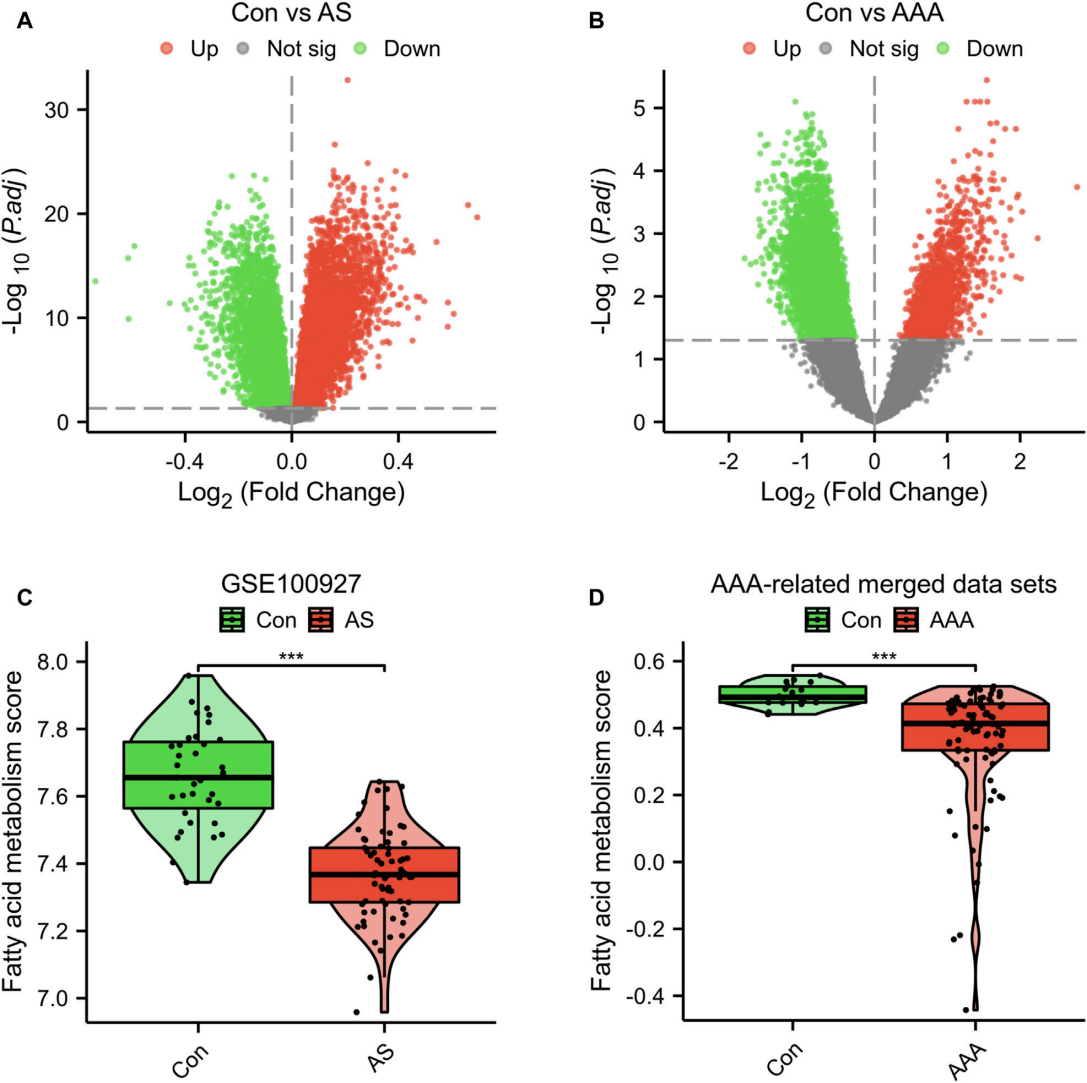
Genetic screening for differential analysis. **(A)** A graph displaying the volcano plot of DEGs in the GSE100927 dataset. **(B)** A graph displaying the volcano plot of DEGs in the AAA-related merged dataset. Comparison of fatty acid metabolism scores in the GSE100927 dataset **(C)** and AAA-related merged dataset **(D)**. ***p < 0.001.

In the dataset GSE100927, which is associated with AS, a total of 13,913 DEGs were identified. Among these DEGs, 6,456 were upregulated and 7,457 were downregulated (Figure 1A). In the dataset related to AAA, a total of 7,890 DEGs were identified. Out of these DEGs, 1,430 showed upregulation while 6,460 showed downregulation (Figure 1B). Furthermore, the GSVA algorithm was utilized to compute the fatty acid metabolism score, revealing a significant decrease in the disease group’s score compared to the Con group (Figures 1C, D). Furthermore, there is a significant reduction in the fatty acid metabolism score in advanced AS compared to early-stage AS (Supplementary Figure S1). These findings suggest that the progression of AS and AAA is closely linked to the regulation of fatty acid metabolism. Consequently, our study proceeded to investigate FRGs in subsequent research.

### Identification of common genes and pathways in both AS and AAA

**FIGURE 2:**
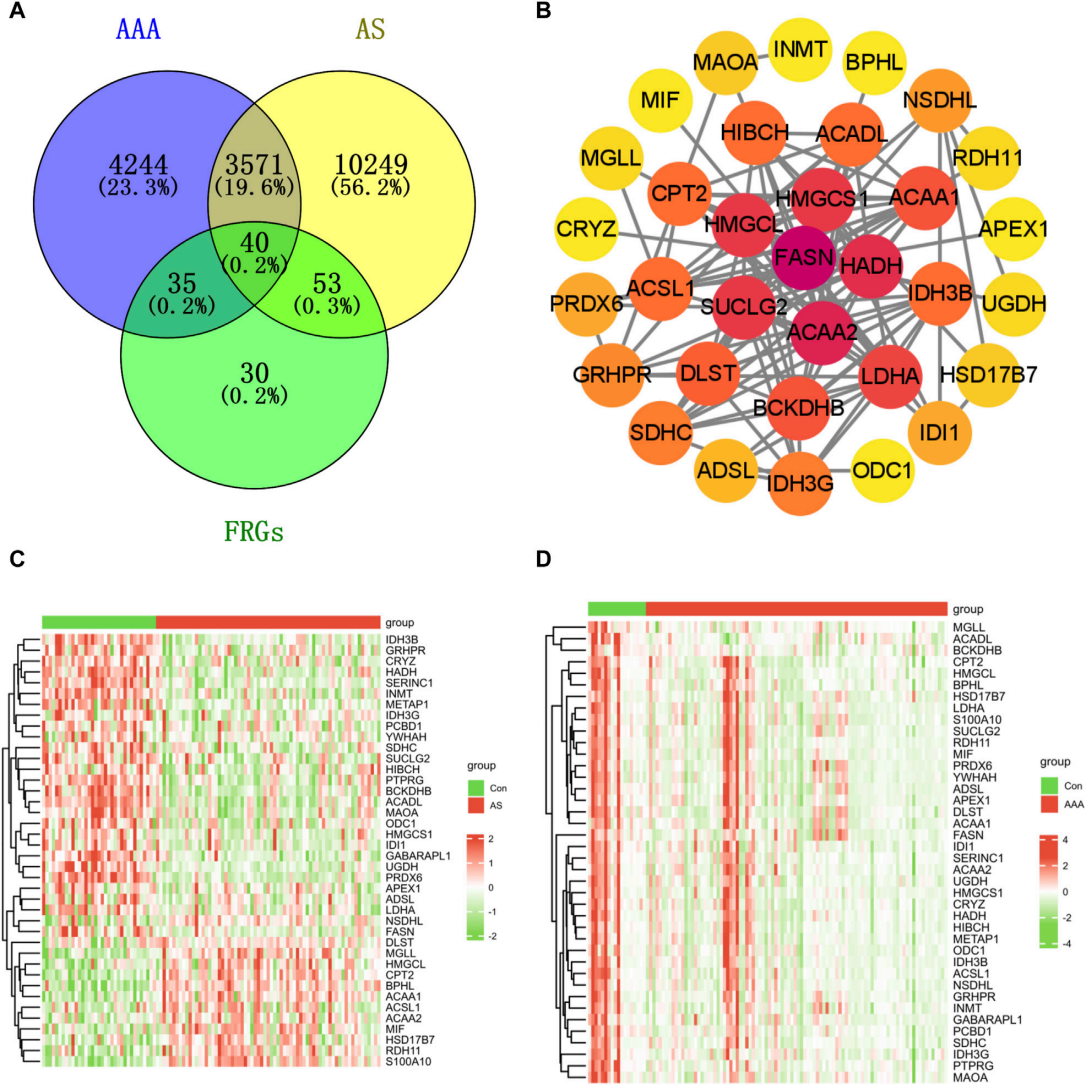
Identification of shared genes in both the AS and AAA datasets. **(A)** The Venn diagram illustrates that there is an overlap of 40 DFRGs in both the AS and AAA datasets. **(B)** The PPI network of shared DFRGs. The heatmap revealed the presence of 40 FRGs between the disease and control groups in the GSE100927 dataset **(C)** and AAA-related merged dataset **(D)**.

A Venn diagram analysis revealed that there were a total of 40 shared DFRGs when comparing AS-related DEGs and AAA-related DEGs (Figure 2A). To explore the potential connections between proteins encoded by the common DFRGs of AS and AAA, the PPI network was visualized using Cytoscape. This network consisted of 33 nodes and 118 edges (Figure 2B). Furthermore, it is evident from the heatmap analysis that a majority of the DFRGs were downregulated in the AS group (Figure 2C) and advanced AS group (Supplementary Figure S2). Specifically, a subset of genes appeared significantly upregulated in the AS group, including DLST, MGLL, HMGCL, CPT2, BPHL, ACAA1, ACAA2, HSD17B7, MIF, RDH11, and S100A10. Similarly, nearly all of the DFRGs showed downregulation in the AAA group when compared to the Con group (Figure 2D).

**FIGURE 3:**
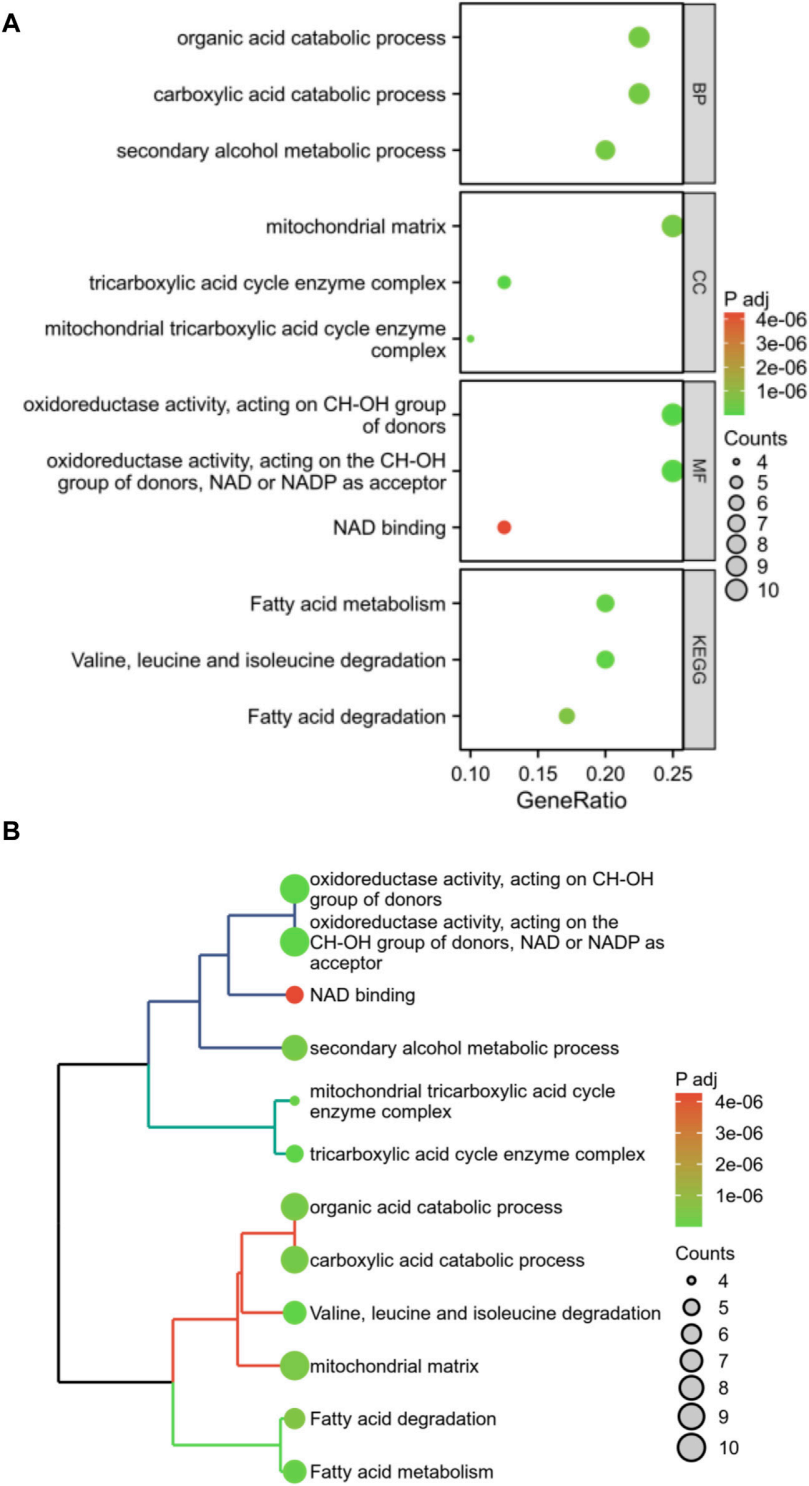
Enrichment analysis of shared DFRGs. The results of enrichment analyses are presented in the form of bubble plots **(A)** and clustered tree plots **(B)**.

Enrichment analysis was conducted on these 40 DFRGs, which are potentially associated with the development of AS and AAA. The results of the GO analysis indicate that these genes might play a role in the organic acid catabolic process, tricarboxylic acid cycle enzyme complex, NAD binding, etc; KEGG analysis revealed that these genes were involved in fatty acid metabolism, fatty acid degradation, etc (Figure 3). Hence, we put forward a daring hypothesis that the occurrence of AS and AAA could be influenced by the pathways associated with fatty acid metabolism.

### The discovery and verification of signature genes

**FIGURE 4:**
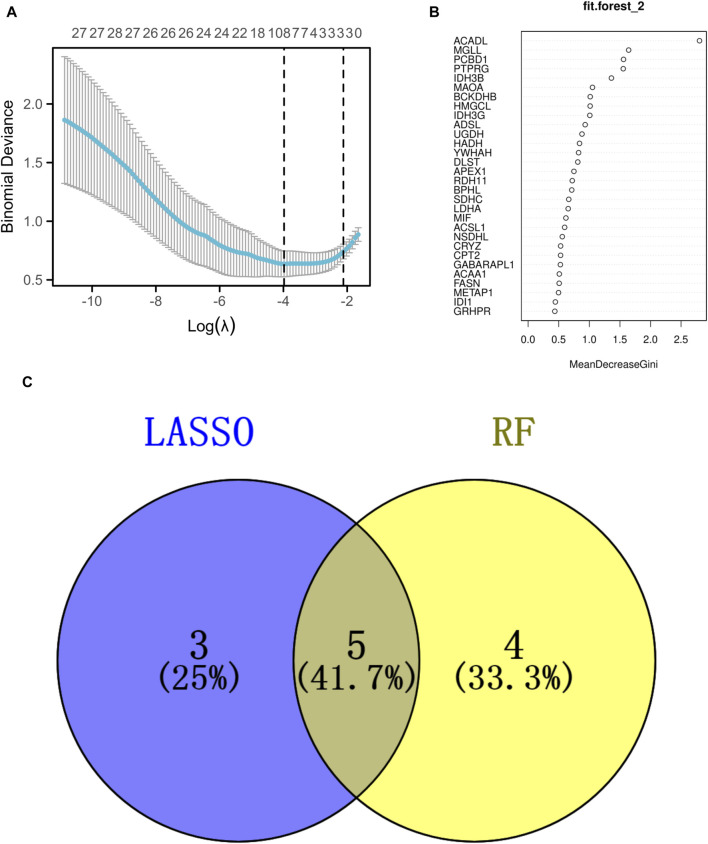
Identification of the signature genes. **(A)** The LASSO regression model was employed to identify shared diagnostic genes. **(B)** The utilization of the RF aimed at identifying common diagnostic genes. **(C)** The identification of five shared diagnostic genes was illustrated through the aforementioned two machine learning techniques, as shown in the Venn diagram.

The analysis utilizing LASSO pinpointed eight pivotal genes as depicted in Figure 4A, while the RF approach revealed nine key genes with a relative importance exceeding 1, as shown in Figure 4B. A Venn diagram, presented in Figure 4C, highlighted the common genes identified by both RF and LASSO methods. Within this shared subset, five genes (PCBD1, ACADL, MGLL, BCKDHB, and IDH3G) were selected for further analysis and validation.

**FIGURE 5:**
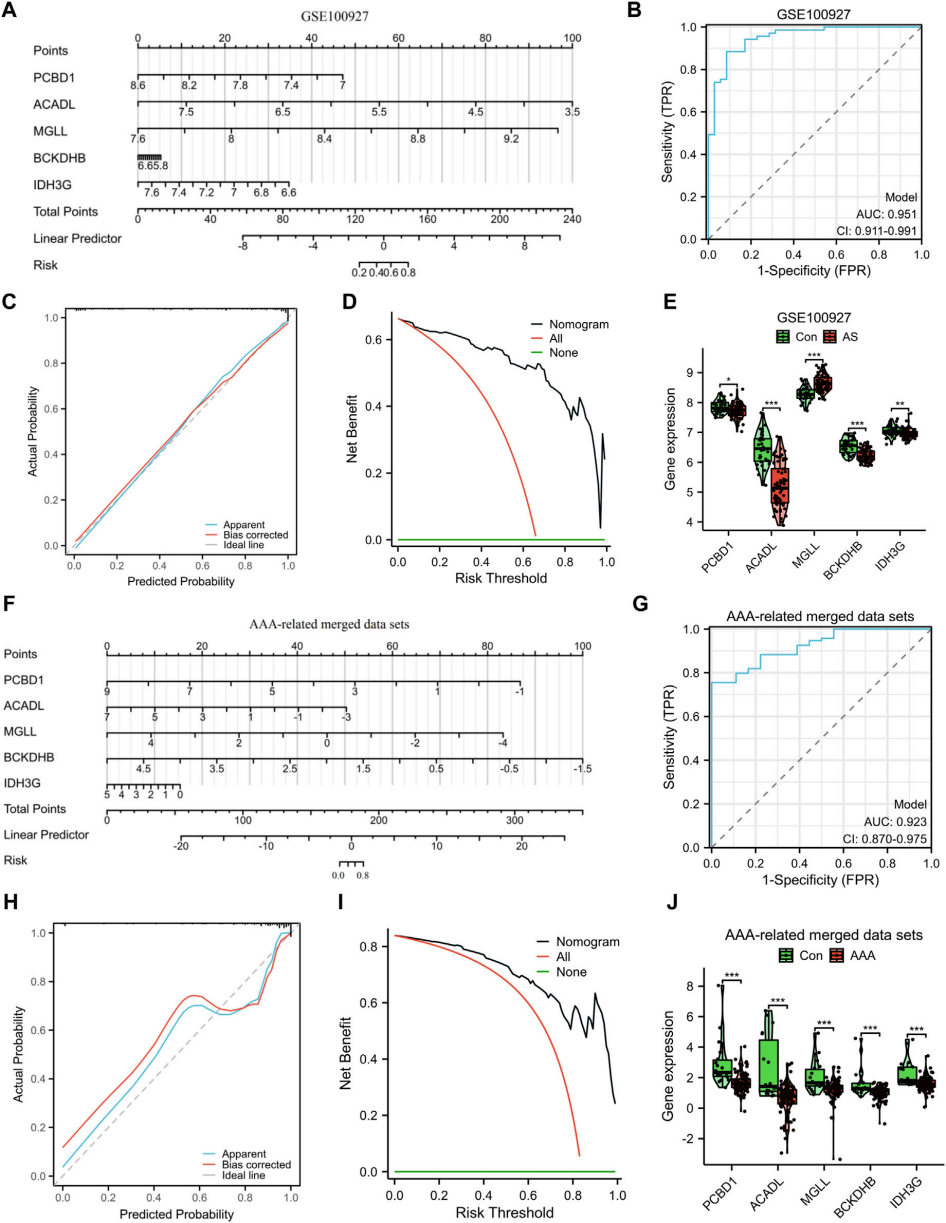
Construction and assessment of a nomogram model. The utilization of a nomogram was employed to evaluate the likelihood of developing AS **(A)** and AAA **(F)**. The ROC curve of the nomogram model for diagnosing AS **(B)** and AAA **(G)**. Calibration curves were used to evaluate the predictive performance of the nomogram model using the GSE100927 dataset **(C)** and AAA-related merged dataset **(H)**. Decision curve analysis curves were employed to evaluate the clinical utility of the nomogram model using the GSE100927 dataset **(D)** and AAA-related merged dataset **(I)**. The comparison of signature genes expression using the GSE100927 dataset **(E)** and AAA-related merged dataset **(J)**. *p < 0.05, **p < 0.01, ***p < 0.001.

To improve the precision of predicting the progression of AS, a nomogram has been created, which considers five specific genes (Figure 5A). By analyzing the receiver operating characteristic (ROC) curve, the model demonstrated a significant area under the curve (AUC) value of 0.95 (Figure 5B). The results from the calibration curve further validated the remarkable precision of the nomogram model in predicting outcomes for patients with AS (Figure 5C). Additionally, the DCA demonstrated the potential advantages of employing the nomogram model for AS patients (Figure 5D). Figure 5E depicts the expression levels of the five signature genes. In the AS group, the expression values of PCBD1, ACADL, BCKDHB, and IDH3G were found to be downregulated, whereas the expression value of MGLL was upregulated. Similarly, utilizing these five characteristic genes, we developed a nomogram specifically for AAA patients. The results obtained from the nomogram were largely in agreement with the findings described earlier (Figures 5F–J).

**FIGURE 6:**
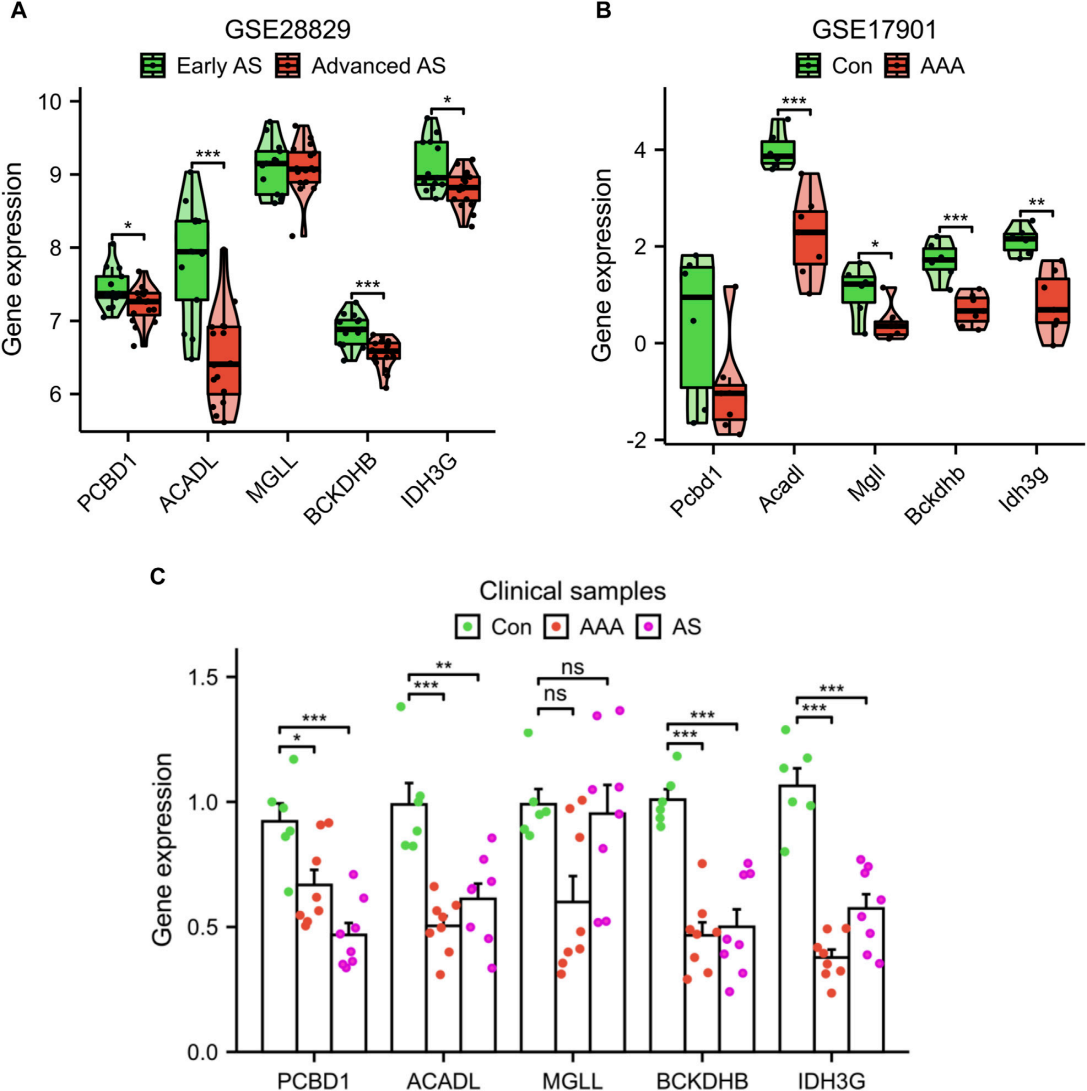
Validation of signature genes expression using the GSE28829 dataset **(A)**, GSE17901 dataset **(B)**, and clinical samples **(C)**. *p < 0.05, **p < 0.01, ***p < 0.001.

Additionally, we confirmed the expression of the signature genes in two separate external datasets (GSE28829 and GSE17901) as well as in clinical samples. The expression levels of PCBD1, ACADL, BCKDHB, and IDH3G genes were observed to decrease in the advanced AS group compared to the early AS group, as depicted in Figure 6A. Similarly, in GSE17901 (Figure 6B), the expression levels of ACADL, MGLL, BCKDHB, and IDH3G genes were found to decrease in the AAA group compared to the Con group. Furthermore, the results of clinical samples demonstrates a decrease in the expression levels of PCBD1, ACADL, BCKDHB, and IDH3G genes in the AAA and AS groups compared to the Con group (Figure 6C).

### Interaction analysis of shared signature genes

**FIGURE 7:**
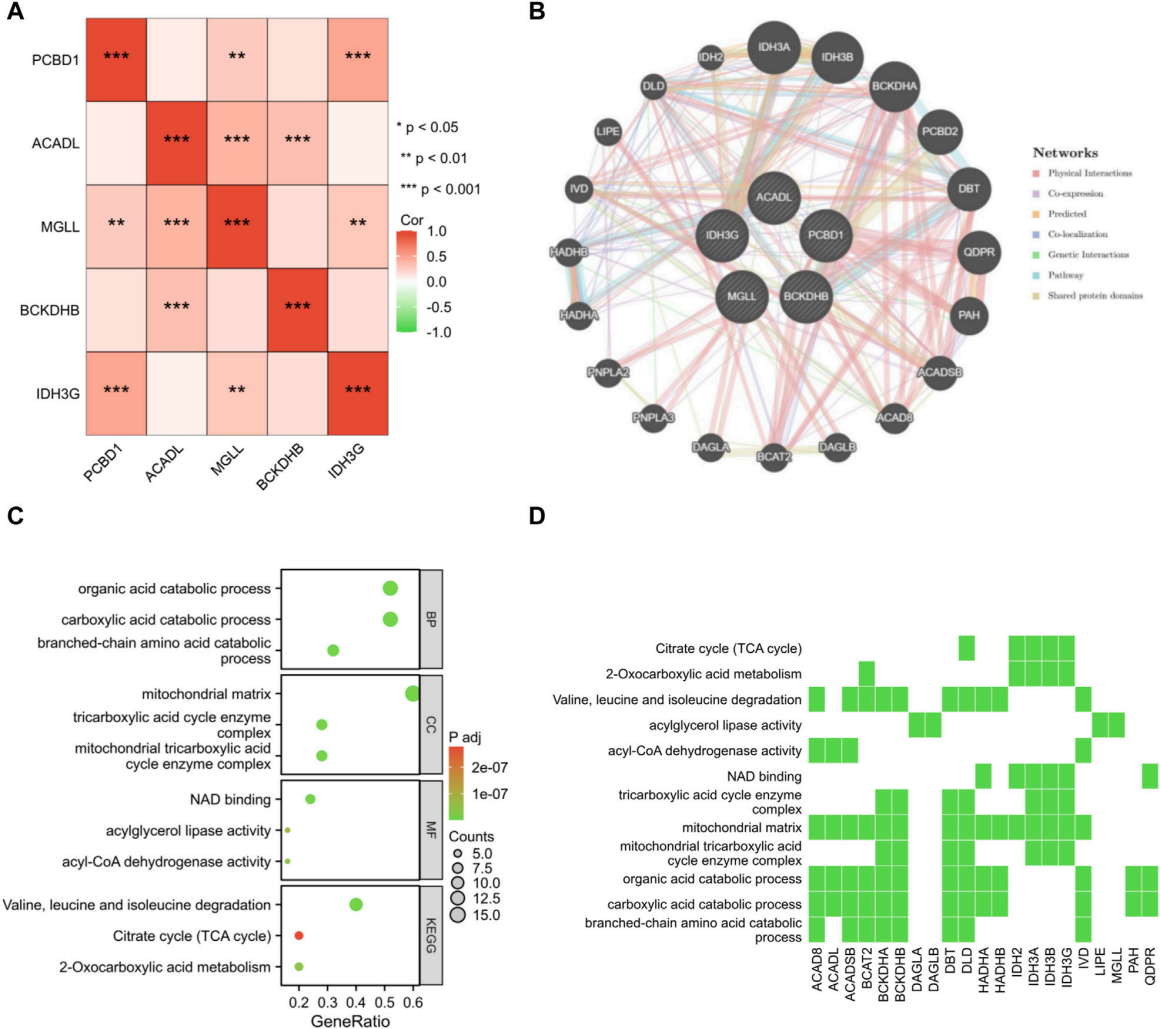
Interaction analysis of shared signature genes. **(A)** Exploring the interconnections among signature genes. **(B)** Mapping the co-expression network of signature genes. Enrichment analysis results are presented in the form of bubble plot **(C)** and heat map **(D)**.

A correlation analysis was performed on the set of five genes that serve as signatures. Remarkably, a positive correlation was observed in the association between PCBD1, IDH3G, and MGLL genes (Figure 7A). By employing the GeneMANIA database, a protein interaction network was constructed for the signature genes, and a total of 20 interacting genes were identified (Figure 7B). GO enrichment analysis showed that these 25 genes were primarily enriched in the organic acid catabolic process, mitochondrial matrix, NAD binding, etc; KEGG enrichment analysis showed that these genes significantly associated with citrate cycle, two-oxocarboxylic acid metabolism, etc (Figures 7C, D).

### Immune infiltration analysis

**FIGURE 8:**
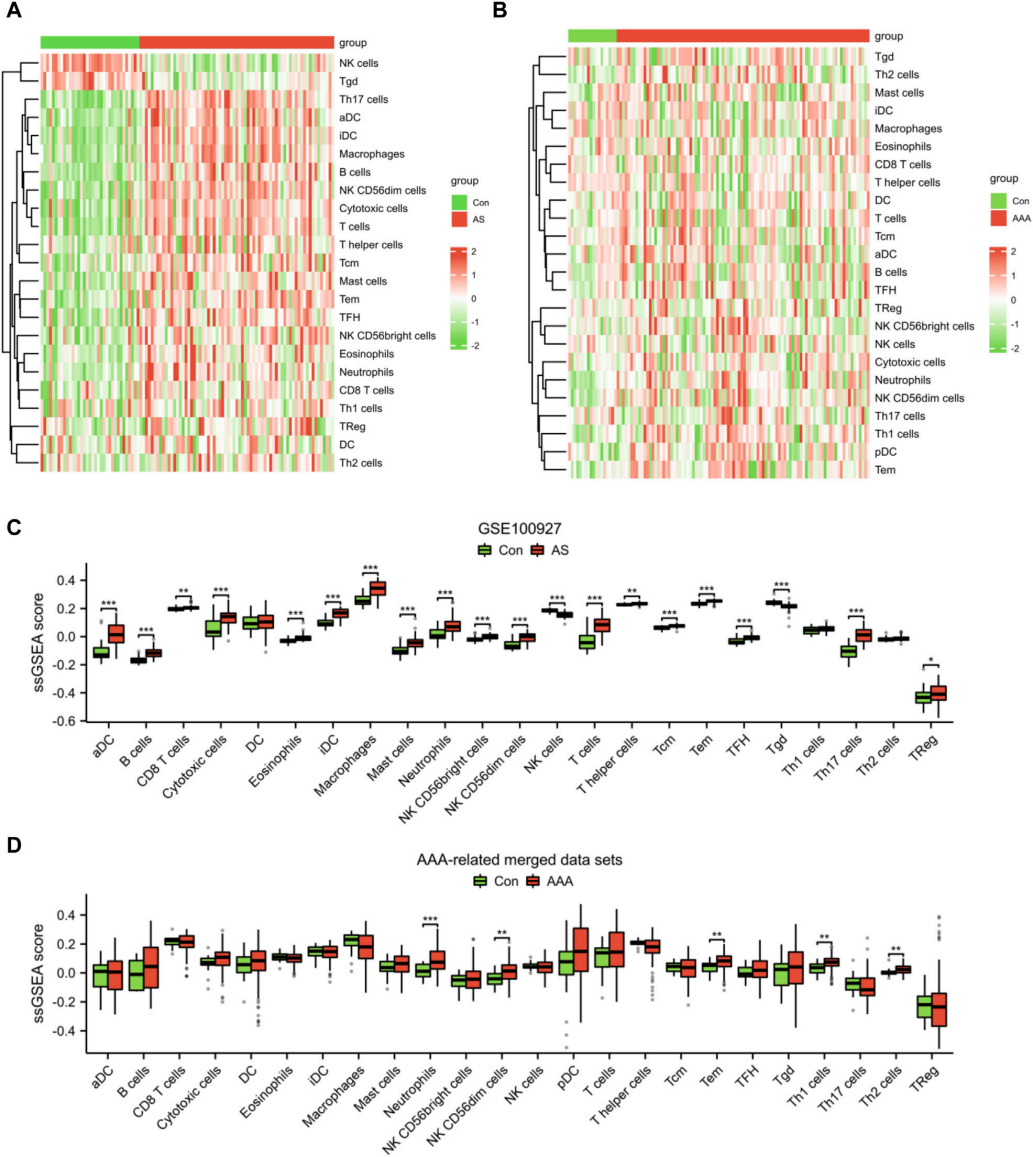
Analysis of immune cell infiltration in AS and AAA samples. Heatmap illustrating the infiltration of 23 different types of immune cells in the datasets related to AS **(A)** and AAA **(B)**. **(C)** The immune cell infiltration between the AS and Con groups can be visualized using a box plot. **(D)** The immune cell infiltration between the AAA and Con groups can be visualized using a box plot. *p < 0.05, **p < 0.01, ***p < 0.001.

Given the significance of immune and inflammatory responses in the progression of AS and AAA, we conducted an immune infiltration analysis using the ssGSEA algorithm. The heatmaps revealed a significant disparity in the distribution of 23 immune cells between the AS samples and the AAA samples (Figures 8A, B). In comparison to the Con group, the AS group exhibited elevated levels of aDC, B cells, CD8 T cells, cytotoxic cells, eosinophils, iDC, macrophages, mast cells, neutrophils, NK CD56bright cells, NK CD56dim cells, T cells, T helper cells, Tcm, Tem, TFH, Th17 cells, and TReg. Conversely, the AS group demonstrated reduced levels of NK cells and Tgd (Figure 8C). Compared to the Con group, the AAA group exhibited elevated levels of neutrophils, NK CD56dim cells, Tem, Th1 cells, and Th2 cells (Figure 8D). It is noteworthy that there was a more substantial increase in the infiltration of neutrophils, NK CD56dim cells, and Tem in both the AS and AAA groups.

**FIGURE 9:**
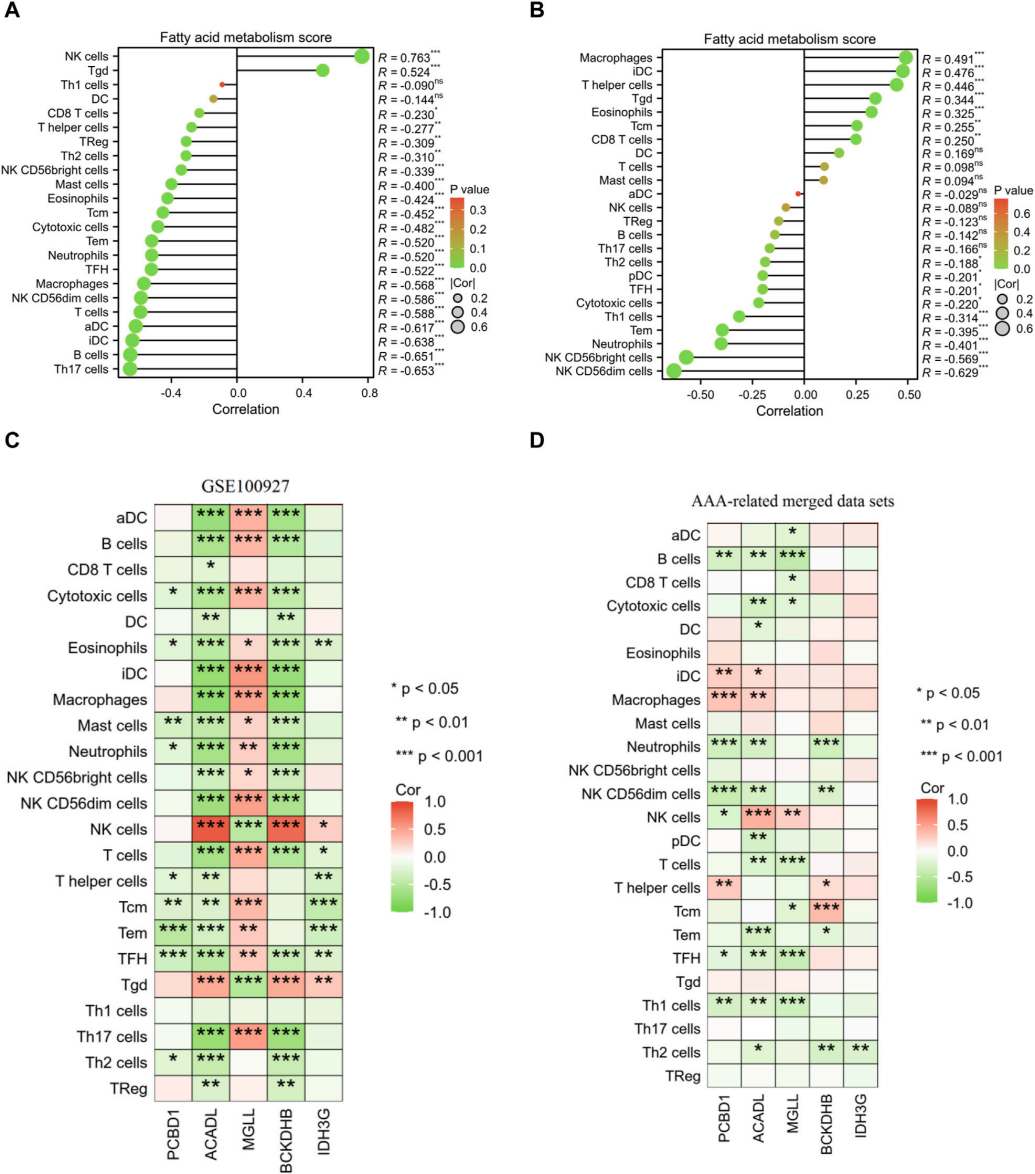
Pearson correlation analysis. Correlation analysis was conducted to examine the relationship between the fatty acid metabolism score and the presence of infiltrating immune cells in samples of AS **(A)** and AAA **(B)**. Correlation analysis was performed to investigate the association between the expression of signature genes and the infiltration of immune cells in AS **(C)** and AAA **(D)** samples.

Furthermore, the results of correlation analysis demonstrated a negative association between the fatty acid metabolism score and the levels of NK CD56dim cells, TFH cells, neutrophils, Tem cells, cytotoxic cells, NK CD56bright cells, and Th2 cells in both AS and AAA samples. Conversely, there was a positive correlation observed between the fatty acid metabolism score and Tgd cells in both AS and AAA samples (Figures 9A, B). The correlation analysis of immune cells with signature genes revealed that TFH and eosinophils exhibited a negative correlation with PCBD1, ACADL, BCKDHB, and IDH3G in AS samples. Similarly, Th1 cells, TFH, and B cells displayed a negative correlation with PCBD1, ACADL, and MGLL in AAA samples (Figures 9C, D).

## Discussion

Numerous studies have consistently shown that patients with AS are at an increased risk of developing AAA (Ito et al., 2008; Trollope and Golledge, 2011; Takahashi, 2021). Yet, the exact relationship between AS and AAA is not fully understood. It is hypothesized that this link may involve chronic inflammatory processes, the death of vascular smooth muscle cells, and the breakdown of the extracellular matrix (Peshkova et al., 2016). These factors are similarly involved in the development of atherosclerotic plaques, which implies that AS, often impacting the aneurysm wall, plays a significant role in the susceptibility to AAA. The imbalance of pro- and anti-inflammatory factors in the context of lipid metabolism issues plays a crucial role in the development and progression of AS (Kotlyarov and Kotlyarova, 2022). There is also a suggestion that AS is a process characterized by the lipid peroxidative stress and buildup of lipids (Borgia and Medici, 1998). In addition, the Mendelian randomization analyses provided evidence to suggest that lipids have a significant impact on the development of AAA (Harrison et al., 2018). Hence, understanding the mechanisms in which lipid metabolism initiates AS and AAA is crucial in the development of novel therapeutic approaches aimed at alleviating the impact of these conditions. In this study, we performed a transcriptomic analysis to identify shared diagnostic biomarkers and explore the immune relationship between AS and AAA based on the fatty acid metabolism gene set. Our findings provide valuable insights into the molecular mechanisms underlying these two pathologies and highlight potential targets for therapeutic interventions.

Firstly, our results demonstrated that 40 FRGs involved in fatty acid metabolism were differentially expressed in both AS and AAA samples compared to healthy controls. Moreover, the GSVA algorithm was employed to calculate the fatty acid metabolism score, which indicated a significant reduction in the score of the disease group as compared to the Con group. These findings suggest that dysregulation of fatty acid metabolism may play a crucial role in the development and progression of both diseases. Previous studies have shown that altered lipid metabolism contributes to the formation of atherosclerotic plaques and the weakening of the aortic wall, leading to the development of AS (Zhu et al., 2016; Guo et al., 2019). The induction of AAA through experimentation leads to a significant change in the metabolic profile of both the aortas and blood, primarily focusing on the modification of lipid metabolism (Guo et al., 2020; Chao de la Barca et al., 2022). In addition, SIRT4 in mitochondria regulates fatty acid oxidation and its deficiency promotes AS through NF-κB pathway activation (Chang et al., 2023). ApoC2, a key member of the apolipoprotein C family, plays a crucial role in activating lipoprotein lipase. Deficiency in ApoC2 leads to severe high triglyceride levels and develops spontaneous AS (Gao et al., 2020). Our findings further support the involvement of fatty acid metabolism in the pathogenesis of these two related conditions.

Furthermore, we have identified five shared signature genes associated with lipid metabolism, namely, PCBD1, ACADL, MGLL, BCKDHB, and IDH3G, which consistently displayed dysregulation in both AS and AAA samples. Furthermore, we opted to validate the signature genes using the mouse AAA model-related dataset (GSE17901), known for its representation of human AAA characteristics, particularly inflammatory and immune processes crucial for aneurysm advancement. Recognizing the limitations of mouse models in mimicking human AAA complexity, especially in systemic lipid metabolism, we gathered clinical samples to further explore the identified genes’ roles and confirm their relevance to AAA and AS pathological features, including alterations in lipid metabolism. Mutations in the PCBD1 gene have been identified as the underlying cause of transient neonatal hyperphenylalaninemia and primapterinuria (Ferrè et al., 2014). A previous investigation presented genetic proof that mutations in PCBD1 can lead to the development of early-onset nonautoimmune diabetes, which exhibits characteristics resembling dominantly inherited HNF1A-diabetes (Simaite et al., 2014). The activation of the TNF signaling pathway is facilitated by ACADL, leading to the promotion of intramuscular adipocyte differentiation (Li et al., 2023). ACADL deficiency pronounced hypoglycemia, accumulation of lipids, elevated levels of free fatty acids in the bloodstream, and impaired insulin sensitivity in the liver (Zhang et al., 2007). ACADL plays a significant role in the unfavorable prognosis and controls the development of breast cancer (Hill et al., 2011). Moreover, ACADL has been linked to the advancement of esophageal squamous cell carcinoma and the negative prognosis of affected individuals (Yu et al., 2018). ACADL was identified as a critical gene in the development of liver hepatocellular carcinoma and is strongly associated with favorable prognosis (He et al., 2022). The crucial factor determining the anti-atherogenic phenotype of dKO mice was found to be the dysregulation of ACADL (Längst et al., 2022). MGLL, an essential metabolic enzyme, performs the crucial function of converting triglycerides into free fatty acids, thus playing a vital role in lipid metabolism (Zhang et al., 2021). The overexpression of MGLL is key in endometrial adenocarcinoma onset and progression, and resistance to progesterone. Inhibitors targeting MGLL hold promise in treating progesterone-resistant endometrial adenocarcinoma (Ma et al., 2022). MGLL, as a lipid metabolic enzyme, is directly linked to the progression of gastrointestinal stromal tumors due to its correlation with adverse clinicopathological (Li et al., 2016). The confirmation of maple syrup urine disease diagnosis involves identifying pathogenic variants in the BCKDHB gene (Strauss et al., 1993). The malignant progression of lung cancer is driven by the promotion of glycolysis-related lactate production and the lactylation process affecting IDH3G (Wang et al., 2023). In the current study, these five signature genes hold promise as potential targets for early diagnosis and monitoring AS and AAA progression. Nevertheless, further research should focus on validating the diagnostic utility of these biomarkers in larger patient cohorts.

Research has indicated that both AS and AAA diseases exhibit an immune response and inflammatory activity involving various types of immune cells (Rizas et al., 2009; Vallejo et al., 2021). The promotion of smooth muscle cells’ synthetic and proinflammatory phenotype is facilitated by neutrophil extracellular traps, thereby contributing to the formation of AAA (Yang et al., 2023). Activation of neutrophils and inflammation of blood vessels result in the formation of a unique microenvironment within the wall of the AAA, characterized by the presence of pro-inflammatory and chemotactic cytokines (Klopf et al., 2021). The impact of neutrophil extracellular traps on the development and advancement of atherosclerotic lesions is undeniable and substantial (Döring et al., 2017). Neutrophil extracellular traps contribute to the inflammation of macrophages and hinder the resolution of AS in diabetic mice (Josefs et al., 2020). In line with these studies, our findings uncovered distinct immune relationships between AS and AAA. Moreover, both the AS and AAA diseases demonstrated a significant rise in the infiltration of neutrophils. Moreover, correlation analysis of immune cells with signature genes revealed specific cell types that were negatively correlated with certain genes involved in fatty acid metabolism. For instance, TFH and eosinophils exhibited a negative correlation with PCBD1, ACADL, BCKDHB, and IDH3G in AS samples, while Th1 cells, TFH, and B cells were negatively correlated with PCBD1, ACADL, and MGLL. These findings suggest potential immune-mediated mechanisms underlying the dysregulation of fatty acid metabolism in AS and AAA.

Despite the progress made in our study, there are still limitations that need to be addressed. Firstly, it is imperative to utilize genetic interference techniques to control the expression of the identified genes in cell culture models. Further research is necessary to clarify the precise role of immune cells in disease pathogenesis and to explore potential immunotherapeutic strategies. In addition, in future research efforts, it is recommended that blood samples be collected from patients with AAA or AS to confirm the presence of these shared diagnostic indicators to facilitate the clinical diagnostic process.

## Conclusion

In conclusion, our transcriptomic analysis identified shared diagnostic biomarkers and unveiled the immune relationship between AS and AAA based on the fatty acid metabolism gene set. These findings contribute to our understanding of the molecular mechanisms underlying these two pathologies and provide potential targets for future therapeutic interventions. Continued research in this field will help advance our knowledge of the complex interplay between lipid metabolism, immune response, and cardiovascular diseases, ultimately leading to improved diagnostic strategies and treatment options for patients with AS and AAA.

## Data Availability

All data used in the present study were available from the GEO database (https://www.ncbi.nlm.nih.gov/geo/).
