# Supplementary material for: Transcriptomic analysis identifies the shared diagnostic biomarkers and immune relationship between Atherosclerosis and abdominal aortic aneurysm based on fatty acid metabolism gene set
Source: Front Mol Biosci. 2024 Apr 10;11:1365447. doi: 10.3389/fmolb.2024.1365447 (PMC11040089; doi:10.3389/fmolb.2024.1365447)
Supplement: Supplementary file 1 [file DataSheet1.docx]

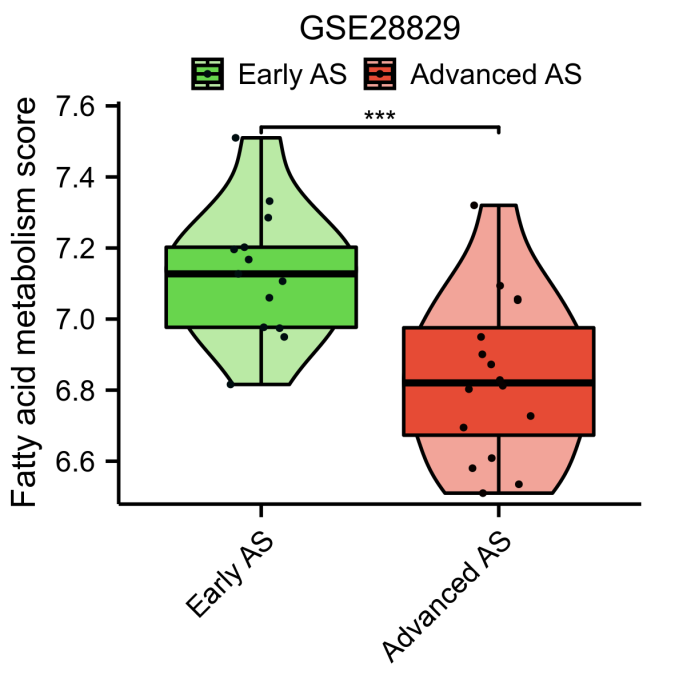


Figure S1 Comparison of fatty acid metabolism scores in the GSE28829 dataset. ***p < 0.001.


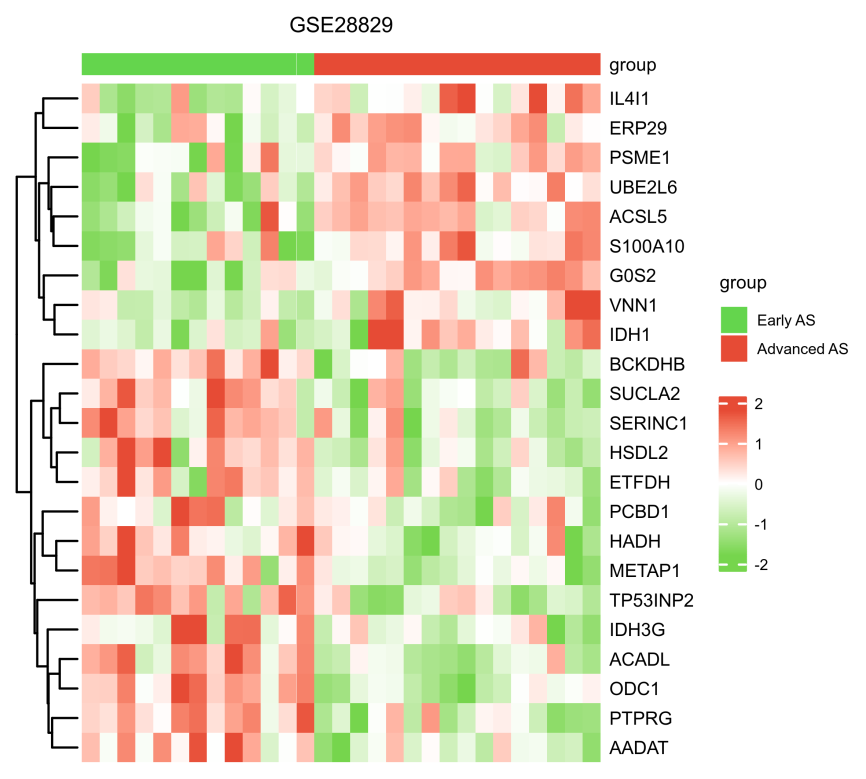


Figure S2 The heatmap revealed the presence of FRGs between the early AS and advanced AS groups in the GSE28829 dataset.

Table S1 Patient characteristics for AAA samples in the GEO database.

| Characteristic | GSE57691 |  |  | GSE47472 |  | GSE98278 |
| --- | --- | --- | --- | --- | --- | --- |
|  | Small AAA | Large AAA | Control | Large AAA | Control | Large AAA |
| Number | 20 | 29 | 10 | 14 | 8 | 31 |
| Aortic diameter (mm) | 53.4±2.3 | 68.4±14.3 | 25.7±1.2 | 62.6±18 | N/A | 62.3±12.1 |
| Sex | Women, 0 (0%) | Women, 2 (7%) | Women, 4 (40%) | Women, 3 (21%) | Women, 3 (38%) | Male, 30 (97%) |
| Age (years) | 68.8±6.9 | 70.5±7.1 | 68.4±4.5 | 71.1±6.2 | 66.8±2.9 | 69.5±7.2 |
| Peripheral arterial disease | 3 (15%) | 5 (17%) | N/A | 1 (7%) | N/A | N/A |
| Hypertension | 15 (75%) | 25 (86%) | N/A | 10 (71%) | N/A | 29 (93.5%) |
| Diabetes mellitus | 4 (20%) | 7 (24%) | N/A | 1 (7%) | N/A | 10 (32.3%) |
| Dyslipidemia | 15 (75%) | 20 (69%) | N/A | 10 (71%) | N/A | 24 (77.4%) |
| Coronary heart disease | 8 (40 %) | 17 (59%) | N/A | 7 (50%) | N/A | 18 (58.1%) |
| Ever smoker | 7 (35%) | 21 (72%) | N/A | 8 (57%) | N/A | 18 (58.1%) |
| BMI (kg/m2) | 28.8±3.2 | 26.8±4.0 | 22.7±9.6 | 28.7±4.6 | N/A | 27.2±3.3 |

Table S2 Baseline characteristics of participants.

| Variables | Normal (n = 6) | AS (n = 8) | AAA (n = 8) |
| --- | --- | --- | --- |
| Age, years | 51 (48-58) | 61 (51-72) | 59 (52-71) |
| Male, n (%) | 4 (66.6%) | 3 (37.5%) | 4 (50%) |
| SBP, mmHg | 121 (114-128) | 142 (129-152) | 144 (125-155) |
| DBP, mmHg | 70 (65-75) | 88 (78-96) | 86 (77-93) |
| TC, mmol/L | 4.25 (3.3-5.3) | 6.59 (5.82-6.9) | 6.83 (5.71-7.21) |
| TG, mmol/L | 1.2 (0.6-1.6) | 1.84 (1.68-1.96) | 1.82 (1.65-1.94) |
| LDL-C, mmol/L | 2.25 (1.36-3.31) | 3.86 (3.58-4.1) | 4.33 (3.6-5.62) |
| HDL-C, mmol/L | 1.9 (1.15-2.21) | 0.83 (0.63-1.1) | 0.75 (0.41-1.23) |
